# The Role of* H. pylori* CagA in Regulating Hormones of Functional Dyspepsia Patients

**DOI:** 10.1155/2016/7150959

**Published:** 2016-10-20

**Authors:** Wang-Ping Meng, Zhong-Qiong Wang, Jia-Qi Deng, Yi Liu, Ming-Ming Deng, Mu-Han Lü

**Affiliations:** ^1^Department of Gastroenterology, The Affiliated Hospital of Southwest Medical University, Luzhou, Sichuan Province 646000, China; ^2^School of Foreign Languages, Southwest Medical University, Luzhou, Sichuan Province 646000, China

## Abstract

*Helicobacter pylori* (*H. pylori*, Hp) colonizes the stomachs of approximately 20%–80% of humans throughout the world. The Word Healthy Organization (WHO) classified* H. pylori* as a group 1 carcinogenic factor in 1994. Recently, an increasing number of studies has shown an association between* H. pylori* infection and various extragastric diseases. Functional dyspepsia (FD) is considered a biopsychosocial disorder with multifactorial pathogenesis, and studies have shown that infection with CagA-positive* H. pylori* strains could explain some of the symptoms of functional dyspepsia. Moreover, CagA-positive* H. pylori* strains have been shown to affect the secretion of several hormones, including 5-HT, ghrelin, dopamine, and gastrin, and altered levels of these hormones might be the cause of the psychological disorders of functional dyspepsia patients. This review describes the mutual effects of* H. pylori* and hormones in functional dyspepsia and provides new insight into the pathogenesis of functional dyspepsia.

## 1. Introduction

Functional dyspepsia (FD), a very common condition that impairs quality of life, is a relapsing and remitting disorder with various chronic symptoms referable to the gastroduodenal region, including typical abdominal bloating or pain, early satiety, belching, heartburn, and nausea in the absence of organic or metabolic disease [1]. There are several diagnostic categories for FD based on the Rome III criteria, which are epigastric pain syndrome (EPS), postprandial discomfort syndrome (PDS), and a combination of these symptoms. The pathogenesis of FD is considered to be multifactorial or even a biopsychosocial disorder that causes abnormal gastrointestinal motility, visceral hypersensitivity, vagal dysfunction, and probable central nervous system disturbance [2]. Currently,* Helicobacter pylori* (*H. pylori*, Hp) infection is considered the major cause of the chronic gastric inflammation of FD patients [3]. Gastric inflammation has been shown to affect motor function and visceral sensitivity in experimental models.* H. pylori* strains that express CagA may be responsible for the FD associated with the more severe forms of gastritis. It was reported that CagA-positive* H. pylori* strains induced more dyspeptic symptoms than CagA-negative or* H. pylori*-negative strains in patients with FD [4]. Some of these functional symptoms can be explained by a gut-driven brain disorder [5], and in vivo hormonal changes have also been implicated. However, no definite clinical manifestation is linked to CagA-positive* H. pylori* strains infection or fluctuating levels of hormones in FD patients. This review discusses the possible correlation between an infection with CagA-positive* H. pylori* strains and the levels of several hormones in FD patients.

## 2. CagA-Positive* H. pylori* Strains-Related Diseases (Figure 1)

### 2.1. Gastric Diseases

Infection with* H. pylori* is recognized as the greatest risk of chronic gastritis, peptic ulcers, mucosa-associated lymphoid tissue (MALT) lymphoma, and gastric adenocarcinoma.* H. pylori* secrete effector molecules to control the inflammatory, proliferative, and apoptotic processes of localized cells. Cytotoxin-associated protein A (CagA) is injected directly into the host epithelial cells via the type-four secretion system (T4SS), which is encoded by the Cag pathogenicity island (PAI) of* H. pylori* type I strains and associated with the development of gastric cancer. In the case of gastric MALT lymphoma, CagA was translocated into B-lymphoid cells and promoted their proliferation, possibly through the CagA-mediated proteins SHP-2, ERK, and MAPK, and increased the levels of Bcl-2 and Bcl-xL [6].

The relationship between infection with CagA-positive* H. pylori* strains and a higher risk of peptic ulcers and gastric adenocarcinoma in humans is widely known [7]. CagA is phosphorylated by host kinases, which alters cell signaling and various cellular responses involved in inflammation. Multiple oncogenic pathways were activated by CagA, such as the Ras/Erk, PI3K/Akt, and Wnt/beta-catenin pathways. Infection with CagA-positive* H. pylori* strains is the main factor driving the hyperactivity of the PI3K/Akt signaling pathway in gastric cancer, which is due to CagA-induced activation of the PI3K/Akt pathway, the representative downstream MEK/ERK pathway, and the nuclear factor-kappaB (NF-kB) signaling pathway, which subsequently induces the nuclear translocation of beta-catenin [8]. As observed in human gastric mucosae infected by CagA-positive* H. pylori*, CagA activates the Wnt/beta-catenin signaling pathway and induces beta-catenin transcription [9]. These cellular responses and inflammation may be responsible for the increase in the level of p53. The level of this key tumor suppressor is increased following infection with CagA-positive* H. pylori* strains and decreased rapidly during* H. pylori* eradication [10], whereas a continuous bacterial infection caused a persistently high level of p53. This phenomenon may be driven by the DNA damage related to inflammatory processes [11].

In addition,* H. pylori* is known to activate the NF-kB signaling pathway. IkappaB kinase alpha (IKK alpha) is a critical regulator of NF-kB activity and* H. pylori* induces the nuclear translocation of IKK alpha, which is indispensable for an inflammatory response, through a Cag PAI-dependent manner [12]. A study explained that CagA could activate the NF-kB signaling pathway and induced the downstream release of IL-8 via the MEK/ERK signaling pathway [13]. Infection with CagA-positive* H. pylori* strains promotes inflammatory processes that result in neoplastic transformation [14]. The inflammatory response associated with CagA-positive* H. pylori* gastritis is due to the upregulated expression of proinflammatory cytokines, including tumor necrosis factor (TNF)-*α*, interferon gamma, interleukin- (IL-) 1beta and IL-8, and, particularly, IL-17, a key cell element in the inflammation caused by* H. pylori*, which mediates the activation of polymorphonuclear neutrophils [15]. Furthermore, IL-17 generally causes IL-8 secretion through the ERK 1/2 MAP kinase pathway [16]. Both CagA protein and Cag PAI have been shown to activate the NF-kB signaling pathway, which increased the level of IL-8 expression, but the effect of CagA in regulating NF-kB activation is still unclear. Recently, a study demonstrated that NF-kB activation and IL-8 release require the Cag PAI-encoded T4SS [17].

### 2.2. Extragastric Diseases

*H. pylori* has also been linked to some extragastric diseases, including cardiovascular, neurological, hematologic, metabolic, and dermatologic disorders, such as nonalcoholic fatty liver disease (NAFLD), gallbladder cancer, colorectal polyps, dental caries, metabolic syndrome, idiopathic thrombocytopenic purpura (ITP), iron deficiency anemia (IDA), coronary artery disease (CAD), and Parkinson's disease (PD) [18].

*H. pylori* eradication was shown to increase the platelet count in patients with ITP. The pathogenesis of ITP due to* H. pylori* infection is probably associated with variable host immune responses to VacA and CagA [19]. Recent guidelines indicated that IDA patients should be evaluated for an* H. pylori* type I strain infection because this bacterium can induce IDA through several mechanisms [20]. Active hemorrhaging caused by CagA-positive* H. pylori* gastritis or ulcers is known to be due to CagA increasing the level of transferrin, thus affecting iron acquisition [21]. Tamer et al. [22] have suggested that* H. pylori* may cause atherogenesis through persistent inflammation, and another possibility for this connection is the molecular mimicry of CagA. The persistence of serum CagA antibodies now appears to be predictive of Parkinson's disease with a poor prognosis; the proposed possible mechanism by which* H. pylori* causes this pathology is that it triggered mitochondrial damage and autoimmunity [23]. Moreover,* H. pylori*-like DNA is more commonly found in liver samples from chronic liver disease patients than from controls [24]. An* H. pylori* infection is positively correlated with metabolic syndrome and is inversely correlated with morbid obesity and type 2 diabetes mellitus (T2DM). The rate of seropositivity is higher in patients with metabolic syndrome than in healthy subjects [25]. Another study demonstrated that more than 75% of gallbladder cancer patients and 50% of chronic cholecystitis patients harbored* H. pylori* in the bile and gallbladder. An* H. pylori* infection was shown to aggravate gallbladder mucosal lesions and even lead to a potentially precancerous condition [26].* H. pylori*-based gastritis is responsible for a higher risk of colorectal polyps, particularly dysplastic adenomas [27]. The findings showed that root canals may be a reservoir for* H. pylori* and a potential source for its transmission [28]. Whether dental plaque is a primary source of* H. pylori* infection of the gastric mucosa of patients with poor oral hygiene needs to be confirmed [29].

The external manifestations of* H. pylori* infections suggest that the mechanism through which* H. pylori* CagA causes diseases is complex and diverse rather than a single pathophysiological mechanism.

### 2.3. Functional Dyspepsia

*H. pylori* is generally accepted as the main pathological agent for the occurrence of functional dyspepsia [30].* H. pylori* CagA protein is associated with the development of functional gastrointestinal disorders (FGIDs). Although the role of CagA in peptic ulcer disease and gastritis is established, its role in functional dyspepsia is controversial. FD patients who are* H. pylori* positive have no clear clinical manifestations and the effect of* H. pylori* eradication is contradictory in these patients. A retrospective study showed that patients with CagA-positive* H. pylori* strains have a higher symptom score and more dyspeptic symptoms than patients with CagA-negative or* H. pylori*-negative functional dyspepsia [31].

### 2.4. Hormone Level Changes after* H. pylori* Colonization

**Figure 2:**
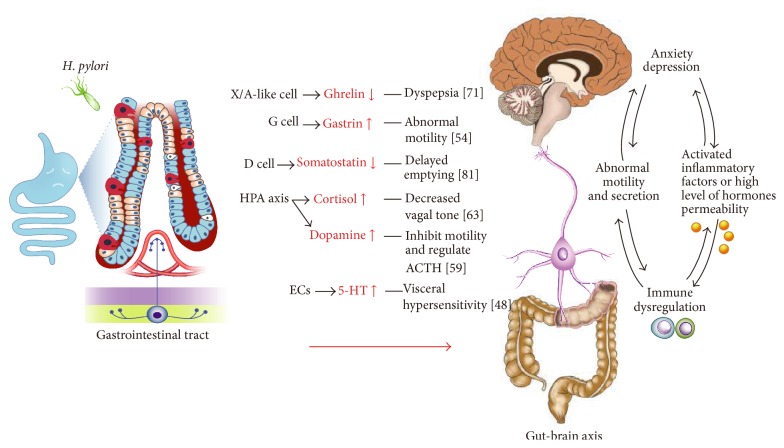
Effects of gastrointestinal hormones level to mental disorders after* H. pylori* colonization. CagA-positive* H. pylori* strains could induce fluctuations in the levels of serotonin (5-HT), ghrelin, dopamine, and cortisol, and might be the cause of some dyspepsia symptoms and mental disorders through blood circulation and brain-gut axis.

An* H. pylori* infection could induce fluctuations in the levels of serotonin (5-HT), ghrelin, dopamine, cortisol, and other hormones in the circulatory system, resulting in damage to various systems, including the central nervous system, and the occurrence of the corresponding symptoms (Figure 2).

### 2.5. 5-Hydroxytryptamine (5-HT, Serotonin)

Psychological disorders, such as anxiety or depression, have been reported to be associated with FD [32]. The results of a population-based investigation suggested that anxiety worsens the symptoms of FD [33]. A 12-year prospective population-based study found that people with higher levels of depression were significantly more likely to develop FD after 12 years [5]. Social anxiety disorder is associated with an overactive presynaptic serotonin system, increased serotonin synthesis, and increased transporter availability. The study by Harmer provided the insight that serotonin pathways may influence the mood of patients with depression by altering how the brain appraises emotional information at an implicit level [34].

Increasing evidence supports a close relationship between 5-hydroxytryptamine (5-HT, serotonin) and gastrointestinal motility and visceral hypersensitivity. Serotonin is synthesized through tryptophan hydroxylase-1 (TpH1) and TpH2, which are found in EC cells and neurons, respectively, and is inactivated by its uptake into enterocytes or neurons via the serotonin reuptake transporter (SERT). Approximately 90% of 5-HT in the body is synthesized in the gut, which has 14 different 5-HT receptor subtypes. The 5-HT 3A receptor and the 5-HT 2A receptor are associated with dyspeptic symptoms, while 5-HT4 receptor agonists may improve dyspeptic symptoms, particularly delayed gastric emptying [35]. In Japan, a 5-HT transporter gene polymorphism was found to be associated with dyspepsia [36].

Abnormal levels of 5-HT have been reported in irritable bowel syndrome (IBS) patients. Raised plasma 5-HT levels were particularly found in female IBS-diarrhea patients, whereas reduced levels were found in IBS-constipation patients. Ahern [37] demonstrated that FD patients had significantly lower preprandial and postprandial plasma serotonin levels compared with those of healthy subjects. Decreased 5-HT levels may impair gastric accommodation or cause visceral hypersensitivity. A significant relationship between postprandial plasma serotonin levels and postprandial dyspepsia scores has been observed, which also indicated that serotonin plays a role in dyspeptic-symptom pathogenesis [37]. However, the role of 5-HT in regulating gastrointestinal tract function is imperfectly understood. Functional dyspepsia (FD) and IBS have been proposed to have a common pathogenesis. Nevertheless, little is known about the role of 5-HT in FD, which is largely due to the presence of various types of 5-HT receptors in the gastrointestinal tract and the absence of suitable and selective antagonists.

Serotonin has been demonstrated to affect many immunological processes and to increase or decrease the levels of proinflammatory cytokines [38]. Chemokines and signaling pathways active during inflammatory processes can affect the synthesis and degradation of serotonin. Stone and Darlington [39] discovered that NF-kB signaling pathway activation increased the rate of release of 5-HT via the phosphorylation of TPH1. It was known that IL-1, IL-2, IL-6, and IFN caused the degradation of the precursor of tryptophan (TRP) through activating an enzyme (indoleamine 2,3-dioxygenase, IDO) and that a decreased level of TRP in the blood leads to a decreased level of 5-HT in the brain. The NF-kB signaling pathway that is involved in gene transcription participates in regulating inflammatory cytokines and plays a key role in the immune response, inflammation, and cell apoptosis in gastrointestinal diseases. Moreover, the increased expression of inflammatory cytokines in turn activates NF-kB. These results suggest a potential relationship between NF-kB activation and the 5-HT level in an infected host.

Because serotonin is a weak platelet agonist, a number of studies have shown an association between severe upper gastrointestinal bleeding with* H. pylori* infection and the levels of selective serotonin reuptake inhibitors (SSRIs). This effect depends on the release of 5-HT by platelets, which acquire serotonin from the blood.* H. pylori* eradication therapy of patients with upper gastrointestinal bleeding was reported to reduce their rebleeding rates [40]. Although 5-HT and* H. pylori* are related to gastric disease, this association requires further studies.

### 2.6. Ghrelin

Ghrelin, which is produced by gastric enteroendocrine X/A-like or G cells and is acylated by ghrelin O-acyltransferase (GOAT) before being released into the blood, plays a crucial role in gastric motility, appetite regulation, and acid secretion. Many gastrointestinal disorders involving inflammation, infection, and malignancy are also associated with altered ghrelin production and secretion. Age, lactation, sex hormones, and the expression of mRNA encoding GOAT, a critical enzyme for ghrelin activity, can impact ghrelin secretion.

A lower level of gastric ghrelin mRNA expression was observed in patients with an* H. pylori* infection compared to that in uninfected subjects [41]. Moreover, due to damage to the gastric X/A-like cells (which produce ghrelin),* H. pylori*-infected patients also have a decreased serum ghrelin level. Inversely, the level of both serum ghrelin and ghrelin mRNA expression can rebound following* H. pylori* eradication therapy, with the consequent relief of dyspeptic symptoms [42, 43]. However, some researchers failed to find significant changes in ghrelin levels after* H. pylori* eradication [44]. They demonstrated that* H. pylori* infection of the stomach did not significantly affect the ghrelin levels. A study of mice found that the normal gut microbiota, independent of* H. pylori*, had a significant effect on ghrelin levels [45]. They also found that the presence of* H. pylori* did not upregulate leptin (a satiety hormone) production or decrease ghrelin secretion in the absence of the normal gastrointestinal microbiota, whereas when* H. pylori* colonized the gut together with the normal microbiota, the opposite result was observed.

An abnormally low level of ghrelin has been found in FD patients, particularly in those with PDS, compared with the level in healthy subjects. And the ghrelin plasma level is associated with the severity of the symptoms in patients with FD [46]. Paoluzi et al. [47] also reported recently that female patients with FD had lower fasting and postprandial ghrelin levels and that the abnormal ghrelin response was apparently involved in their meal-related symptoms. Although whether altered ghrelin levels are the cause or the result of dyspepsia is unclear, these data imply a possible role for ghrelin in the pathogenesis of FD patients with* H. pylori* positive. The gastritis induced by an* H. pylori* infection is predominantly related to T helper (Th)1/Th17 cell immunity. Ghrelin suppresses Th cell-dependent pathology. The downregulated level of ghrelin in the gastric mucosa of* H. pylori*-infected patients might promote an ongoing Th1-cell response and chronic active gastritis [48]. B. L. Slomiany and A. Slomiany [49] reported the role of phosphatidylinositol 3-kinase (PI3K) in digestive tract mucosa infected with* H. pylori*, demonstrating that the modulation of ghrelin as a gastric mucosal response to* H. pylori* depends on PI3K activation. The ghrelin receptor is highly specific, which means that only the acylated form of ghrelin can bind to it, and active ghrelin stimulates the appetite though neuropeptide Y (NPY). Because ghrelin receptor activation leads to the enhanced activity of the NPY pathway, activating the ghrelin receptor would be beneficial in abating early satiety and appetite loss. However, the mechanisms through which ghrelin is regulated in FD patients require more studies.

### 2.7. Gastrin and Somatostatin

Gastrin, CCK, and somatostatin are all sensitive to the stress of anxiety [50]. Gastrin, which is released by G cells in the pyloric mucosa, stimulates gastric acid secretion, promotes cell growth (increasing the rate of cell division and inhibiting apoptosis), and transacts with cholecystokinin-2 receptors (CCK2Rs). The CCK2Rs, mitogen-activated protein kinase 1 (MP1), and ERK1/ERK2 are reported to mediate the gastrin-induced growth of gastric adenocarcinomas [51]. Patients with chronic renal failure have 2- to 3-fold higher serum gastrin levels because the kidneys are responsible for clearing gastrin [52]. Somatostatin is produced by D cells and neurons in the gastrointestinal tract and pancreas. In the stomach, the antrum mucosa secretes acid to stimulate the secretion of somatostatin and the latter inhibits gastrin secretion. That phenomenon explains why PPIs stimulate gastrin secretion by decreasing acid secretion, thus resulting in sustained hypergastrinemia, gastrinoma, and atrophic gastritis and possibly in gastric carcinoid tumors. It was shown that an acute* H. pylori* infection activates the sensory neurons associated with somatostatin stimulation [53].* H. pylori* infection and a reduced somatostatin level have a complex etiological relationship in chronic gastritis. In* H. pylori*-positive patients, a decreased somatostatin content (likely mediated by proinflammatory cytokines) leads to increased gastrin secretion, perhaps due to the effect of* H. pylori* (a direct effect of CagL, a component of the T4SS for CagA) on G cells [54]. A study showed that Cag PAI-positive* H. pylori* strains or CagL activate the gastrin promoter, whereas Cag PAI-negative strains do not [55]. A further study indicated that gastrin expression stimulated by CagL is involved with epidermal growth factor receptor and MP1 signaling.

In investigating the relationship between the gastric motility disorder and the gastrointestinal hormone abnormality in the GI mucosa of FD patients, Van Oudenhove et al. [56] found that the levels of gastrin in the postprandial plasma and the gastric mucosa were significantly higher in FD patients with delayed emptying and suggested that the altered gastrin levels may play a role in the pathophysiology of the abnormal gastric motility of FD patients.

A study found that weight loss and symptom severity in FD patients were determined by somatization and depression [57]. A positive relationship was found between the degree of dyspeptic symptoms and the level of somatostatin [50]. The cited study also indicated that CCK and somatostatin might correlate certain psychological reactions with the pathophysiology of FD.

### 2.8. Dopamine (DA)

The homeostasis of the digestive system is dependent on the activity of aminergic mediators [such as 5-HT, noradrenaline (NA), and dopamine (DA)], which play crucial roles in the central or peripheral generation of gastrointestinal motility, secretion, and sensation. Dopamine is mainly produced by mesenteric organs (the GI tract, spleen, and pancreas) and is metabolized by monoamine oxidase and catechol-O-methyl-transferase (COMT). DA is known to modulate diverse physiological functions of the digestive system, such as acid and mucus secretion in the stomach and bicarbonate excretion in the duodenum. DA exerts its biological functions through two types of receptors, the D1-type receptors (D1 and D5) and D2-type receptors (D2, D3, and D4) receptors. In the digestive system, DA inhibits motility via the D1-type receptor present on smooth muscle and modulates the release of acetylcholine (ACTH) from myenteric neurons via D2-type receptors. Dopamine and its agonist have a protective effect on the development of various lesions in the gastroduodenum. It was also reported that dopamine might protect the gastric mucosa against acidified ethanol through the activation of the *α*2 adrenoceptor, which leads to inhibition of gastric motility [58]. Recently, it was shown that dopamine acts as a strong antitumor/antiangiogenic factor by suppressing the expression of growth factors, such as vascular endothelium-derived growth factor (VEGF), to inhibit angiogenesis in malignancies [59]. However, another study suggested that dopamine is rapidly metabolized by COMT in the gastrointestinal tract; therefore, there would not be a sufficient effect on ulcer healing [60]. Giusti et al. [61] found that dopamine receptor antagonists or inhibitors, such as diazepam and other antipsychotic drugs, can stimulate the development of peptic ulcers and infections by* H. pylori*. However, Kirschbaum and Hellhammer [62] found the rate of* H. pylori* infection was increased after dopamine treatment in women with a high prolactin level. These studies may indicate a fuzzy relationship between dopamine and* H. pylori* infection, the pathological mechanism of which requires further study. Although DA receptors are considered to modulate GI motility and GI motor symptoms associated with FGIDs, their potential role in the pathophysiology of functional dyspepsia is still unknown.

### 2.9. Cortisol

Cortisol, which plays an important role in the defense of a host infected with bacteria, is secreted through the stress-mediated activation of the hypothalamic-pituitary-adrenal (HPA) axis. High levels of adrenocorticotropic hormone (ACTH) and cortisol are generally considered to be outcomes of an HPA-axis disorder. Moreover, the levels of the ACTH immunoreactive substance (IS) are regulated by negative feedback from neurogenic stimulation and plasma cortisol. On the other hand, leptin suppresses HPA-axis activity, whereas ghrelin stimulates neuropeptide Y and food intake, which induces high concentrations of plasma ACTH and cortisol. HPA-axis alterations are related to gut motor functions [63]. Moreover, serum-free cortisol fluctuations are associated with various symptoms of functional gastrointestinal disorders (FGIDs), including FD [64].

Some studies showed that an increased serum cortisol level promoted* H. pylori* colonization. Koşan et al. found that, compared with those of the healthy control group, the serum IGF-I and IGF-II concentrations were significantly decreased in* H. pylori*-positive patients, although their serum cortisol level was increased. These authors did not discuss the probable effect of cortisol on* H. pylori* infections [65]. In contrast, Katagiri et al. [66] reported that* H. pylori*-infected patients had significantly decreased cortisol levels than* H. pylori*-negative patients. They also demonstrated that cortisol prevents* H. pylori* colonization through strengthening the host defense mechanisms. Recently, studies have shown that drugs, such as cimetidine, reduce basal and stimulated cortisol synthesis through inhibiting enzymes. However, proton pump inhibitors, such as lansoprazole and rabeprazole, are thought to cause increased cortisol levels in a starvation condition due to their possible effects of stimulating the HPA axis and increasing the plasma ACTH-IS level [67].

However, few studies have investigated HPA-axis parameters in FD patients, and inconclusive results were obtained. Adults with FD who have an autonomic nervous system disorder have been reported, and the most common finding in these patients is decreased vagal tone [68]. In PDS patients, mental stress before a meal increases the symptom severity through sympathetic hyperactivity and increased cortisol levels. Another study suggested that these neurohormonal responses to HPA-axis activation mainly affect gastric sensitivity [69]. A study in which the HPA-axis activity of FGID patients was inhibited showed that they displayed both salivary morning cortisol and diurnal cortisol levels that were significantly lower than those of controls [70]. De la Roca-Chiapas et al. [64] have reported that FGID patients with high cortisol levels complained of more depression than those with low or medium cortisol levels, whereas the latter described experiencing more pain. In contrast, another study did not find a tendency toward higher cortisol levels in FD patients and suggested that these patients have not had enough exposure to daily stress to activate the HPA axis. It is not impossible that both stress-induced anxiety and an altered neuroendocrine response could increase the severity of dyspeptic symptoms (Table 1).

### 2.10. Treatment of FD

The diverse clinical manifestations and the uncertain pathophysiological mechanisms make it difficult to select a therapeutic strategy to manage FD. There is currently no established treatment regimen. Updated guidelines for FD treatment have been published by academic gastroenterology organizations, such as the American Gastroenterological Association, promoting a comprehensive strategy that includes diet, behavior modification and cognitive therapy, psychological interventions, and drug therapy [71]. However, drug treatment is still the main form of practical FD treatment in China.* H. pylori* eradication and treatment with antacids, motility-regulatory drugs, and antidepressants are commonly recommended as an effective method to treat FD.

Patients in Asian countries receive a relatively higher benefit from* H. pylori* eradication. Researchers have shown that* H. pylori* eradication can result in a long-term remission of FD symptoms [72]. Some evidence suggested that* H. pylori* eradication significantly improved gastrointestinal symptoms in patients with EPS compared with its effects on patients with PDS [73].* H. pylori* eradication certainly has a statistically significant but small benefit in patients with FD. It is likely that the results of* H. pylori* eradication reflect not only the effect of treating an* H. pylori* infection but also the effect on the gastrointestinal microbiome. The results of some randomized controlled trials (RCTs) support eradication therapy [74], whereas most other studies found no benefit. A Cochrane review reported no significant difference between patients who had received a placebo and those who had undergone* H. pylori* eradication. Furthermore, due to the wide use of antibiotics in* H. pylori* eradication regimens, drug-resistant* H. pylori* strains are arising. The success rate of triple-antibiotic eradication therapy is less than 80% throughout the world. This phenomenon makes it challenging for clinicians to manage infections with* H. pylori* strains that are resistant to antimicrobial agents, particularly those that are resistant to clarithromycin and metronidazole. As a second-line treatment, moxifloxacin has been investigated. Zhang et al. [75] suggest that moxifloxacin-based therapy is more effective than the standard triple or quadruple therapy for this disease. However, its adverse effects, such as tendonitis or a nervous system reaction, are a matter of concern. The diverse results of therapy may be due to the use of different trial designs, different methods of patient selection, or different* H. pylori* eradication strategies. Furthermore, accumulating evidence demonstrates that that the human stomach contains a complex microbial ecosystem that does not include* H. pylori* strains [76]. The balance of these communities is crucial for health maintenance, and their disturbance is considered to be involved in gastrointestinal diseases [77]. Andersson et al. [78] reported that the gastric mucosa displayed a diverse microbiota after* H. pylori* eradication, although it is difficult to discriminate whether the changes in the gastric microbiota were caused by* H. pylori* eradication or by antibiotic treatment.

Dyspepsia patients have significantly more health care consultations due to psychological distress. Chronic stress is considered a major risk factor for FD, possibly due to brain-gut axis dysregulation mediated by the hypothalamic-pituitary axis [79]. Antianxiety and antidepressant drugs have been reported to have positive effects on FD, particularly on intractable FD, with tricyclic antidepressants (TCAs) and small doses of selective serotonin uptake inhibitors (SSRIs) most often mentioned as efficacious [72]. When these drugs are used to treat FD, clinicians should consider the direct effects of neurotransmitters on gastrointestinal disorders as well as their effects on mental disorders because they may affect the modulatory function of neurotransmitters (e.g., 5-HT) on gastrointestinal sensory, motor, and secretory functions. However, 5-HT receptors have been recognized as the target points for symptomatic improvement. 5-HT3 receptor antagonists and 5-HT4 receptor agonists have been selected to treat functional diarrhea or constipation. 5-HT4 receptor agonists cause the release of acetylcholine, which stimulates smooth muscle contraction, leading to accelerated gastric emptying [80].

Because the pathogenesis of FD is uncertain and most likely multifactorial, individualized treatment should be established based on the patients' chief complaints.

## 3. Summary

Whether there are distinct modes of pathogenesis of PDS and EPS remains controversial. Fang et al. [81] found that although PDS and EPS have some common risk factors, including a younger age, anxiety, and NSAID consumption, different risk factors appear to be associated with different FD subgroups; for example,* H. pylori* infection, an unmarried status, sleep disturbance, coffee consumption, and depression disorder are risk factors only for PDS but not for EPS. These finding may indicate the distinct etiopathogenesis of the subgroups of FD.

CagA and 5-HT may participate in the pathogenesis of FD, but the specific underlying pathogenic mechanisms are still unclear.* H. pylori* infection and anxiety or depression are also significant factors in the pathogenesis of FD. Therefore, whether and how CagA and 5-HT affect the pathogenesis of FD (including PDS and EPS) are a topic worthy of study and more research is needed. Moreover, the wide distribution of 5-HT receptors and their respective roles in the pathogenesis of FD are of special significance and are worthy of further study.

## Figures and Tables

**Figure 1:**
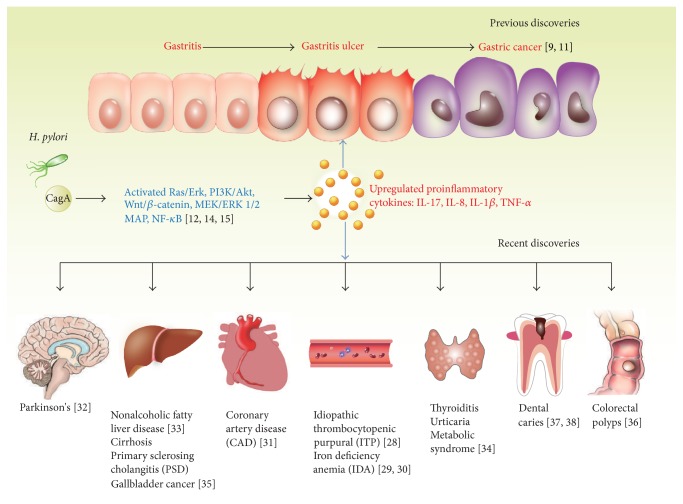
CagA-positive* H. pylori* strains-related diseases. According to previous research findings, infection with CagA*-*positive* H. pylori *strains is widely known as a higher risk of peptic ulcers and gastric adenocarcinoma. However, recent discoveries have shown that* H. pylori* is associated with some extragastric diseases, including cardiovascular, neurological, hematologic, metabolic, and dermatologic disorders. Inflammatory response and multiple signaling pathways might participate in mediating pathophysiological process.

**Table 1:** Possible relationship between *H. pylori* and several hormones.

		Relative enzymes	Possible signaling pathway	Altered hormones	Receptors	Consequence
CagA (+) *H. pylori* strains	To play a role in the regulation of hormones by certain factors such as CagA, CagL, MP1, IL-17, IL-8, or abnormal autonomic nervous system	TPH1	NF-kB	5-HT	5-HTR 2A/3A	Dyspepsia
					5-HTR 4	Delayed gastric emptying
		GOAT	P13K-Akt	Ghrelin	GHSR	Decreased motility Lower acid secretion Anorexia
		Monoamine oxidase or COMT	cAMP ↑ → PKA↑	Dopamine	D1/D5	Psychotic
			AC activation ↓		D2/D3/D4	Gastroduodenal lesion Tumor
		GC-GR compound	HPA axis	Cortisol	GR	Host defense mechanism recedes *H. pylori* colonization
		Cag PAI	MP1 signaling	Gastrin	CCK2Rs	Increased gastric acid Atrophy gastritis
		CCK	HPA axis	Somatostatin	SSTR	Decreased gastric acid Chronic gastritis

TPH1: tryptophan hydroxylase-1; GOAT: ghrelin O-acyltransferase; GHSR: growth hormone secretagogue receptor; COMT: catechol-O-methyl-transferase; AC: adenylate cyclase; GC: glucocorticoid; GR: glucocorticoid receptor.
